# Microchimerism as a source of information on future pregnancies

**DOI:** 10.1098/rspb.2023.1142

**Published:** 2023-08-30

**Authors:** Francisco Úbeda, Geoff Wild

**Affiliations:** ^1^ Department of Biological Sciences, Royal Holloway, University of London, Egham TW20 0EX, UK; ^2^ Department of Mathematics, The University of Western Ontario, London, Ontario, Canada N6A 5B7

**Keywords:** inclusive fitness, parent–offspring conflict, intra-genomic conflict, game theory, pre-eclampsia, autoimmune diseases

## Abstract

Small numbers of fetal cells cross the placenta during pregnancy turning mothers into microchimeras. Fetal cells from all previous pregnancies accumulate forming the mother’s fetal microchiome. What is significant about microchimeric cells is that they have been linked to health problems including reproductive and autoimmune diseases. Three decades after the discovery of fetal microchimerism, the function of these cells remains a mystery. Here, we contend that the role of microchimeric cells is to inform the fetus about the likelihood that its genes are present in future pregnancies. We argue that, when genes are more likely than average to be in future maternal siblings, fetuses will send a fixed number of cells that will not elicit a maternal immune response against them. However, when genes are less likely to be in future maternal siblings, fetuses will send an ever-increasing number of cells that will elicit an ever-stronger maternal immune response. Our work can explain the observed clinical association between microchimeric cells and pre-eclampsia. However, our work predicts that this association should be stronger in women with a genetically diverse microchiome. If supported by medical tests, our work would allow establishing the likelihood of pregnancy or autoimmune problems advising medical interventions.

## 1. Introduction

**Figure 1.**
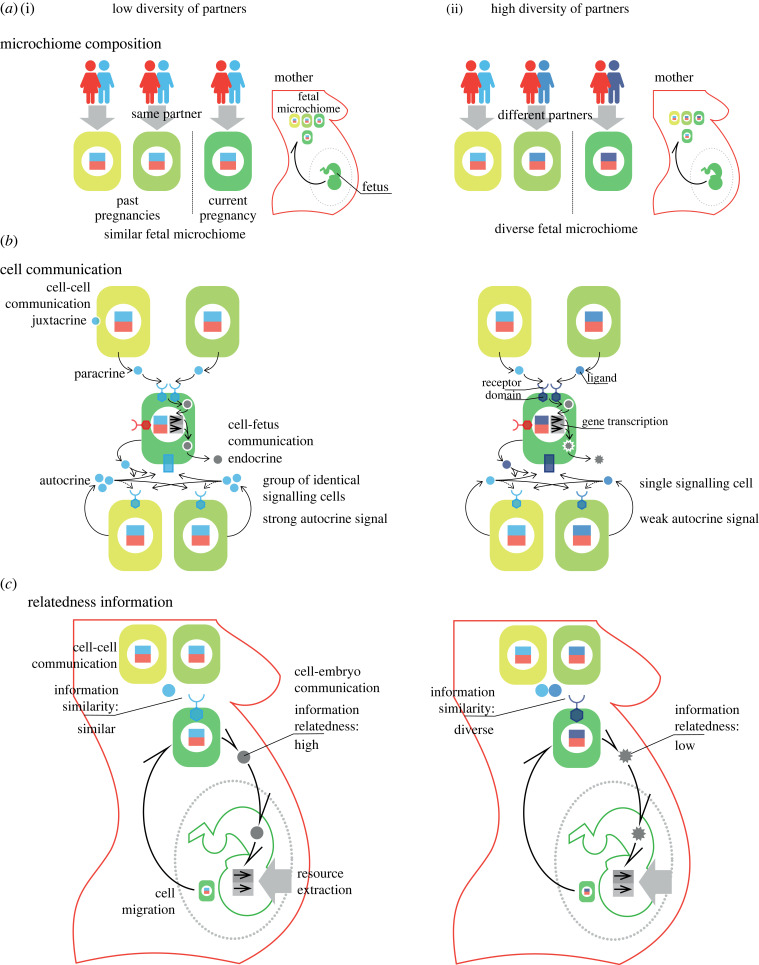
Information and the fetal microchiome. We present a schematic of how (i) lower-than-average and (ii) higher-than-average diversity of mating partners impacts the genomic variability of the fetal microchimeric population. Panel (*a*) illustrates how the genome composition of the microchiome reflects mother’s mating diversity. Panel (*b*) illustrates plausible cell communication mechanisms transmitting information on future relatedness to siblings. Panel (*c*) illustrates cell–fetus communication mechanisms which condition the extraction of resources.

During pregnancy, fetal and maternal cells cross the placenta [1–3] (figure 1). The presence of a relatively small number of fetal cells in mothers that are genetically distinct from their fetus, turns mothers into microchimeras. Fetal microchimerism refers to the fetal cells transferred to the mother and maternal microchimerism refers to maternal cells transferred to the fetus [2,3]. More cells are transferred from fetus to mother than the other way round [4]. In this research, we focus on the role of fetal microchimerism. This pool of cells is often referred to as the fetal microchiome [5].

Interestingly, fetal cells may persist in a mother for her entire life [6]. Therefore, throughout their reproductive lives, mothers accumulate fetal cells from each of their past pregnancies including those resulting in miscarriages (figure 1) [2,3]. Furthermore, mothers inherit, from their own mothers, a pool of cells contributed by all fetuses carried by their mothers, often referred to as grandmaternal microchimerism [2,3,7,8]. As a result, the fetal microchiome in a mother is made up of a pool of cells contributed by her pregnancies and her matrilineal pregnancies across several generations. Mothers have some control over their microchiome; in particular, they have the ability to remove fetal cells via their immune system. Following delivery, fetal cells are targeted by the immune system and their number in the maternal bloodstream is drastically reduced [9]. However, microchimeric cells are not completely eliminated, and their number increases again in the next pregnancy becoming more abundant with gestational age [10].

Fetal microchimerism has been associated with different maternal-health problems, most significantly reproductive health and autoimmune diseases [2,11–13]. Pregnant mothers suffering pre-eclampsia have greater concentrations of fetal michrochimeric cells in the bloodstream [14–16]. Other studies suggest a role for fetal cells in miscarriage and premature birth [17–19]. Greater concentrations of fetal cells in blood have also been associated with some autoimmune diseases including systemic lupus and multiple sclerosis, though there are conflicting results [11,13].

The breadth and significance of maternal-health problems associated with fetal microchimeric cells raises the question, why do mothers tolerate them at all? Possibly, these cells serve some important function, but their role remains an evolutionary mystery.

It has been proposed that the evolutionary role of fetal cells is to extract maternal resources [2,20,21]. Drawing on evolutionary theory, Haig [20,21] and Boddy [2] have suggested that fetal cells may be the result of parent–offspring conflict over resource allocation: whereas a mother is selected to distribute resources equally among their offspring (both present and future), each of her offspring is selected to pursue more than its fair share [22,23]. Thus, Haig [20,21] and Boddy [2] claim that fetal microchimeric cells, being an extension of the fetus, may serve to extract maternal resources for the selfish benefit of the offspring. Of course, mothers in this scenario would be selected to oppose the cells and so their presence in the mother’s bloodstream is thought to reflect a failure of her immune system. Therefore, this *extraction theory* predicts that fetal microchomeric cells should accumulate during pregnancy in those maternal tissues where they can most strongly influence the transfer of maternal resources to the fetus, for example in the mammary glands or the brain [2,20,21,24]. Evidence is inconclusive regarding the distribution and role of fetal microchimeric cells with some found in breast and brain but most of them found in the liver and lungs (organs with limited connections to the transfer of resources) [2].

In contrast to the extraction theory, we suggest that the role of fetal microchimeric cells is to inform the fetus about the likelihood that copies of its genes will be present in future pregnancies (its relatedness to future offspring produced by its mother [25]). Fetal microchimeric cells in mothers carry information about the relatedness of the present fetus to future maternal siblings (figure 1). We assume that the current fetus can access this information by sending cells that interact with other cells from past pregnancies by means of standard cell–cell communication mechanisms [26]. We propose either paracrine or autocrine interactions mediated by genes coding for ligands and receptors of a given allelic type (figure 1). Degrees of binding between ligands and receptors result in the expression of genes that result in the secretion of different ligands into the bloodstream communicating relatedness to the fetus (figure 1). We argue that information about relatedness will impact the offspring’s extraction of resources during pregnancy (see Haig [23] for ways in which the fetus extracts resources from its mother). To formulate our *information theory of microchimeric cells*, we build a model to determine whether fetuses will be selected to send cells to gather information and whether mothers will be selected to keep these cells or remove them. Finally, we make predictions about the genetic diversity of the fetal microchiome in mothers and maternal health.

## 2. Results

Consider a population of mothers that vary with respect to the diversity of their mating partners. At one extreme, mothers may mate with the same partner for their entire reproductive life. At the other extreme, mothers may mate with a different partner every time they reproduce. We want to emphasize that diversity of mating partners may not be active decisions made by mothers, and any consequences that follow from the change in partners should not be understood as value judgements of human behaviour.

We assume that offspring extract resources from their mothers to survive to maturity [22]. The resources extracted, *x*, benefit the offspring extracting those resources, *b*(*x*), at a cost to all future offspring of its mother, *c*(*x*) [22]. The mother’s diversity of mating partners, *s* (henceforth, maternal ‘type’ for short), determines the relatedness between the present offspring and the future offspring produced by the mother. We use *r*(*s*) to denote the extent of this relatedness, where 1/4 ≤ *r*(*s*) ≤ 1. The fitness of a gene encoding an offspring’s extraction of maternal resources is given not only by the resources the offspring takes in but also by the resources the offspring takes away from its maternal siblings, that is, its inclusive fitness [25]. Following Hamilton [25] and Trivers [22], the inclusive fitness of an offspring extracting resources given its mother’s strategy is

2.1 $$f_{o}(x,r(s))=b(x)-r(s)c(x).$$

Because a mother is equally related to each of her offspring (past, present and future), her inclusive fitness [22,25] is

2.2 $$f_{m}(x)=b(x)-c(x).$$

As explained elsewhere [27–30], individuals appear to have been designed to maximize their inclusive fitness. That said, parent–offspring conflict theory assumes that offspring solve this maximization problem without the benefit of information on the type of mother caring for them. Thus, natural selection acts on the offspring’s inclusive fitness with relatedness being the expected one, $\bar{r}$, that is $f_{o}(x,\bar{r})$ [22,31,32]. Offspring are then predicted to extract an optimal amount of resources, say $x_{o}^{*}$, where $x_{o}^{*}$ satisfies $\partial_{x}\;f_{o}(x,\bar{r})=0$. Because $x_{o}^{*}$ will depend on $\bar{r}$, we express it as a function of this quantity, namely $x_{o}^{*}(\bar{r})$. Parent–offspring conflict theory shows that, generically, the optimal amount of resources from the offspring’s perspective is greater than the optimal amount of resources that mothers are selected to give. If $x_{m}^{*}$ is the latter amount, then it must satisfy ∂_*x*_ *f*_*m*_(*x*) = 0, and we have $x_{o}^{*}(\bar{r})>x_{m}^{*}$.

We assume that offspring have information of varying accuracy, *a*, that allows them to estimate the type of mother caring for them. This estimate, in turn, informs the relatedness of the offspring to its future maternal siblings, $\hat{r}(s,a)$. Note that the estimated relatedness changes with the availability of information *a*: less accurate information brings the estimate closer to the expected relatedness $\bar{r}$, while more accurate information brings the estimate closer to the exact relatedness *r*(*s*). Specifically, we model the estimate the offspring makes as

2.3 $$\hat{r}(s,a)=(1-a)\bar{r}+ar(s).$$

The relatedness estimate informs the optimal resource-extraction of the offspring, $x_{o}^{*}\circ \hat{r}(s,a)=x_{o}^{*}(\hat{r}(s,a))$, where this quantity satisfies $\partial_{x}\;f_{o}(x,\hat{r}(s,a))=0$. We assume that $x_{o}^{*}\circ \hat{r}(s,a)$ is the actual amount of resources extracted by the offspring with information *a* from a mother of type *s*.

Now we suppose that the accuracy of information *a* is determined by the number of microchimeric cells, with increased numbers of cells improving accuracy and decreased numbers of cells reducing accuracy. We consider a trait expressed by the offspring that increases the number of microchimeric cells when upregulated. Specifically, we assume *y* is the rate of migration of fetal cells into its mother. We consider another trait expressed by the mother that decreases the number of microchimeric cells when upregulated. Specifically, we assume *z* is the strength of the maternal immune response to the presence of fetal cells. In this scenario, information is a function of the migration rate of fetal cells into the fetus’s mother, and the destruction rate of fetal cells by the maternal immune system, that is, *a* = *χ*(*y*, *z*) with ∂_*y*_*χ* > 0 and ∂_*z*_*χ* < 0.

The inclusive fitness of an offspring in a mother of type *s* when the offspring has access to information *χ* is

2.4 $$w_{o}(y,z)=b(x_{o}^{*}\circ \hat{r}(s,\chi (y,z)))-r(s)c(x_{o}^{*}\circ \hat{r}(s,\chi (y,z))).$$

The inclusive fitness of a mother of type *s* when her offspring has access to information *χ* is

2.5 $$w_{m}(y,z)=b(x_{o}^{*}\circ \hat{r}(s,\chi (y,z)))-c(x_{o}^{*}\circ \hat{r}(s,\chi (y,z))).$$

**Figure 2.**
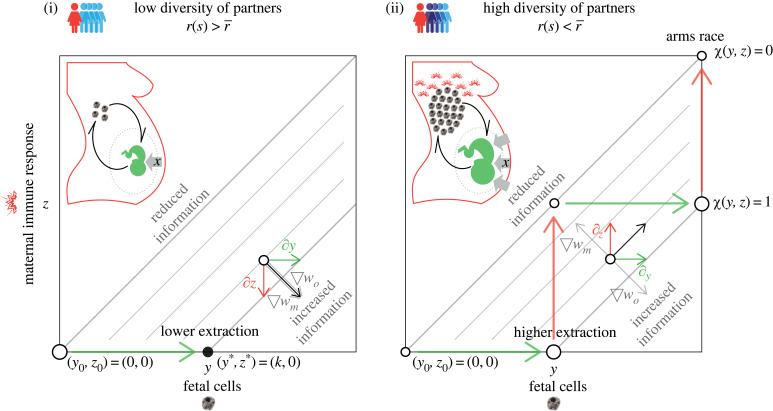
Sketch of information accuracy outcomes when the number of fetal cells and maternal immune response to those cells coevolves. Panel (i) shows coevolution in mothers with lower diversity of mating partners where fetuses are more related to future maternal siblings. Panel (ii) shows coevolution in mothers with higher diversity of mating partners where fetuses are less related to future maternal siblings. Grey lines show combinations of fetal cells, *y*, and maternal immune response, *z*, that produce the same degree of accuracy, *a*, that is, contours of the *χ*(*y*, *z*) surface. Thick grey lines show the particular cases of no information *χ*(*y*, *z*) = 0 and perfect information *χ*(*y*, *z*) = 1. Grey arrows are orthogonal to contours and show the direction in which inclusive fitness increase is greatest ($\nabla w_{o}$ for offspring and $\nabla w_{m}$ for mother). Green arrows show the direction selection drives migration of fetal cells *y*. Red arrows show the direction selection drives maternal immune response to fetal cells *z*. Black arrows point in the net direction of evolution. Dots indicate equilibria and their area the amount of resources extracted. Thick green and red arrows show the best strategy of fetus and mother respectively from the current equilibria. Following dots and thick arrows, we find the coevolutionary outcome. When fetuses are more related than average to future maternal siblings (i) there is no conflict between fetus and mother and the outcome is perfect information from fetal cells that are tolerated by mothers. When fetuses are less related than average to future maternal siblings (ii) there is conflict and the outcome is an arms race between fetus and mother with fetuses sending an ever increasing number of cells and mothers mounting an ever stronger immune response to fetal cells.

**Figure 3.**
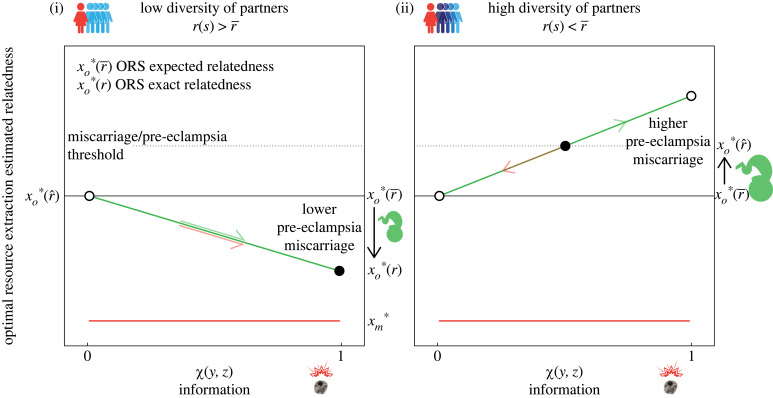
Sketch of resource extraction outcomes when information coevolves in fetus and mother. Panel (i) shows coevolution when fetuses are more related to future maternal siblings. Panel (ii) shows coevolution when fetuses are less related to future maternal siblings. Green lines show the optimal resources extraction (ORS) of maternal resources by the fetus a certain accuracy of the information. Red lines show the optimal resources donation of maternal resources by the mother herself. Green and red arrows show the direction selection drives the accuracy of information, *a* = *χ*(*y*, *z*), in fetus and mother respectively and the optimal resource extraction by the fetus $x_{o}^{*}$. Dots indicate equilibria with empty ones being stable and solid ones unstable. The dashed back line represents a hypothetical threshold of resource extraction that when crossed results in reproductive health problems. When fetuses are more related than average to future maternal siblings (i) there is no conflict between fetus and mother and the outcome is perfect information from fetal cells and lower extraction of resources by the fetus. When fetuses are less related than average to future maternal siblings (ii) there is conflict. If the fetus wins the conflict, the equilibrium lies near the optimal resource extraction with perfect information $x_{o}^{*}(r)$. If the mother wins the conflict however, the equilibrium lies near the optimal resource extraction with no information $x_{o}^{*}(\bar{r})$. When the forces between fetus and mother are balanced, the equilibrium lies between perfect and no information $x_{o}^{*}(r)>x_{o}^{*}(\hat{r})>x_{o}^{*}(\bar{r})$.

We find that, in mothers with lower-than-average diversity of partners, natural selection favours offspring who send cells to their mothers and favours mothers who have a weak immune reaction against microchimeric cells. In other words,

2.6 $$r(s)>\bar{r}\;\text{implies}\;\partial_{y}w_{o}>0\;\text{and}\;\partial_{z}w_{m}<0,$$

and there is no conflict between offspring and mothers with both favouring the presence of microchimeric cells (figure 2). Equation (2.6) tells us that coevolution between offspring and mothers results in offspring sending enough cells for them to reveal its relatedness to future siblings, and mothers who do not mount an immune reaction against microchimeric cells (figure 2). In this coevolutionary outcome, informed offspring extract fewer resources than uninformed offspring, that is, $x_{o}^{*}(\bar{r})>x_{o}^{*}\circ \hat{r}(s,a)=x_{o}^{*}\circ r(s)>x_{m}^{*}$ (figure 3).

In mothers with higher-than-average diversity of partners, natural selection favours offspring who send cells into their mothers and favours mothers who mount a strong immune reaction against microchimeric cells, that is

2.7 $$r(s)<\bar{r}\;\text{implies}\;\partial_{y}w_{o}>0\;\text{and}\;\partial_{z}w_{m}>0.$$

Now there is conflict between offspring and mothers with offspring favouring the migration of fetal cells but mothers favouring their destruction (figure 2). Coevolution between offspring and mothers results in an arms race with offspring sending an ever increasing number of cells, and mothers mounting an ever stronger immune reaction against microchimeric cells (figure 2). In this case, an undetermined amount of resources will be extracted by the offspring. Though undetermined, the amount will lie somewhere between the amount extracted under a perfectly accurate assessment (namely $x_{o}^{*}\circ r(s)$) and a perfectly inaccurate one (namely $x_{o}^{*}(\bar{r})$), that is, $x_{o}^{*}\circ r(s)>x_{o}^{*}\circ \hat{r}(s,a)>x_{o}^{*}(\bar{r})>x_{m}^{*}$ (figure 3). Of course, if $r(s)=\bar{r}$ then both offspring and mother are indifferent to cell numbers.

The previous findings rely on mothers knowing the diversity of their mating partners in their lifetime relative to the average mother. If mothers do not have this information, their inclusive fitness would be the expected inclusive fitness, that is, ${\bar{w}}_{m}(y,z)$. Thus, not knowing the diversity of their lifetime mating partners, mothers will be selected to eliminate microchimeric cells under some circumstances and retain them under others. In the Methods, we show that maternal elimination of cells occurs when fetus exhibits what Faria & Gardner [33] call a ‘convex’ optimal resource extraction in response to kinship information. Specifically, when inclusive-fitness cost is linear or accelerating, a convex response to kinship implies mothers are selected to eliminate cells. We also show that maternal retention of cells can occur when fetus exhibits a ‘concave’ optimal resource extraction in response to kinship information [33]. Specifically, if costs are linear or accelerating, then a mother being selected to retain cells implies a concave response to kinship. In both cases, we can loosely view a mother as using the geometry of the optimal offspring response to encourage them to extract fewer resources, thus bringing their behaviour closer to the maternal optimum.

## 3. Discussion

We propose that the role of fetal microchimeric cells is to inform fetuses about their relatedness to future maternal siblings (figure 1). We find that, in all cases, offspring are selected to send cells that establish and transmit information on their relatedness to future siblings. We find that mothers with lower-than-average partner diversity are selected to retain all cells as means of disclosing the higher relatedness between current and future siblings (figure 2). Mothers with higher-than-average partner diversity, however, are selected to eliminate all cells as a means of concealing the lower relatedness between current and future siblings (figure 2). The information conveyed by microchimeric cells, in turn, conditions the offspring’s extraction of maternal resources. Fetuses informed about their relatedness to future maternal siblings extract fewer resources from mothers with lower-than-average diversity of mating partners, but extract more resources from mothers with higher-than-average diversity of partners (figure 3).

We find that mother and offspring are not necessarily in conflict over the fetal microchiome. Mothers with lower-than-average diversity of partners are not in conflict with their fetus over the presence of microchimeric cells in their bodies. These mothers and their offspring are expected to cooperate in maintaining a cell population that is mutually beneficial. As such, cell migration into the mother should be just enough to provide full information about relatedness to the fetus, and mothers should not mount an immune response against fetal cells (figure 2). The extraction of maternal resources by informed offspring should be lower than the extraction by uninformed ones (figure 3). Mothers with higher-than-average diversity of partners are in conflict with their fetus over the presence of microchimeric cells in their bodies. These mothers and their offspring are expected to fight over a cell population that benefits only the fetus. As such, fetuses are selected to send cells into their mothers and mothers are selected to mount an immune response against them (figure 2). Escalation of this conflict should result in offspring sending increasingly larger amounts of cells and mothers mounting an increasingly stronger immune response (figure 2). The extraction of maternal resources by informed offspring should be greater than the extraction by uninformed ones; exactly how much greater, though, will depend on whether it is the offspring that succeeds by sending enough cells to transmit full information about relatedness despite its mother’s attacks, or it is the mother that succeeds by destroying enough cells before they can transmit information about relatedness (figure 3). Note that our model establishes the existence of a conflict over information (what Godfray [34] calls *battleground models*) but does not investigate resolutions to this conflict (what Godfray [34] calls *resolution models*).

Our model relies on the assumption that the information about relatedness contained by the pool of paternal genomes of the fetal microchiome can be communicated between cells (figure 1). Cell–cell communication is ubiquitous in multicellular organisms [26]. Here, we discuss two plausible mechanisms of communication that would achieve the goal of informing cells sent by the fetus about relatedness. The first mechanism relies on paracrine (or juxtacrine [26]) interactions between fetal cells sent by the current embryo and resident cells in the fetal microchiome. Migrating cells produce a standard receptor molecule, consisting of an extracellular domain that can bind ligands produced by other cells, and a cytoplasmic domain that can initiate actions within cells, including the expression of genes (figure 1(ii)). Resident cells in the microchiome produce signalling molecules that may bind receptors expressed in the current-pregnancy cells. Together, allelic variants of ligands and receptors with higher affinity for each other when produced by cells carrying the same alleles would constitute a mechanism for future-kin discrimination (figure 1(ii)). Finally, binding between ligand and receptor would lead to the expression of genes in the migrating cells that produce a new signal that is, in turn, secreted into the bloodstream and targets the fetus (standard endocrine interaction with the fetus) (figure 1(iii)). The greater the similarity between the fetal microchiome and current-pregnancy cells, the stronger the signal sent to the embryo.

The second mechanism relies on autocrine [26] interactions between fetal cells sent by the current embryo and resident cells in the fetal microchiome. In this case, both migrating and resident cells produce both receptor and ligand molecules. In autocrine communication, ligands can bind not only receptors in neighbouring cells but also receptors in their own cell membrane (figure 1(ii)). Ligands produced by current-pregnancy cells could start an autocrine response by binding the receptors of neighbouring microchimeric cells. A microchiome consisting of a single allelic variant would then produce a strong autocrine signal with high concentration of ligands. By contrast, a microchiome consisting of multiple allelic variants would produce a weak autocrine signal with low concentration of ligands (figure 1(ii)). Receptors in the current-pregnancy cells able to detect the concentration of ligands would constitute a mechanism for future-kin discrimination (figure 1(ii)). Finally, detection of high (or low) concentration of ligands would lead to the expression of genes in the migrating cells that produce a new signal that is secreted into the bloodstream and targets the fetus (figure 1(iii)). The greater the similarity of the fetal microchiome, the stronger the signal sent to the embryo. Interestingly, autocrine signalling is particularly active in embryonic and cancer cells [26]. The mechanism we propose here is related to quorum sensing mechanisms [35]. Note that our model is valid not only for the two cell communication mechanisms discussed here but for any other mechanism able to convey relatedness to future siblings.

It is known that the number of microchimeric cells in maternal blood increases with gestational age [10]. Thus, the possibility of interaction between cells migrating from the current fetus and resident cells from previous pregnancies increases with gestational age. Unlike previous work, our theory does not predict the presence of microchimeric cells in any particular organ but in any organs where the possibility of interaction between cells is highest.

In our model, migrating cells find a fetal microchiome in mothers. One could argue that this is not true for the first fetus conceived. Note that it should be the first conceived because miscarriages leave fetal cells behind [18] and thus a first born offspring may have interacted with a fetal microchiome. While the first fetus conceived does not find a fetal microchiome, it does find a grand-maternal microchiome [8]. Mothers inherit from their own mothers a grand-maternal microchiome that contains information on the diversity of mating partners of the grandmother, the grandmother’s mother, and so on. If the mating diversity of the current offspring’s mother is partly determined by the mating diversity of her matrilineal ancestors, the first conceived offspring has in the grand-maternal microchiome the source of information it needs to discriminate future kin. Significantly, there is evidence in support that mating diversity is, partially, heritable [36–40]. The greater the heritability the more accurate the information the fetus can extract from the grand-maternal microchiome.

In a similar fashion, early fetuses have less accurate information that later fetuses. The first fetus relies on the grand-maternal microchiome. Subsequent fetuses rely on the information left by previous conceptions that add to the grand-maternal microchiome. The greater the number of conceptions preceding the fetus considered, the greater the accuracy of the information that the fetal microchiome can provide. However, our model works for any level of accuracy as long as microchiomes provide information that improves the estimated relatedness. In that sense, the qualitative predictions of our model do not change with conception order. However, we do expect it to make a difference in resolution models where the amount of fitness gain is relevant to determine the resolution of the conflict.

We consider current offspring and mother as the only parties involved in microchimeric interactions. There is a third party, however, corresponding to past offspring that left cells behind to be used as information. It could be argued that the focus we place on a mother and its current offspring unduly disregards the perspective of past offspring whose cells also contribute to the microchiome. However, the interests of cells belonging to past offspring are aligned with those of the mother in that, like the mother, they are equally related to all future offspring. Thus, we have not unduly disregarded key parts of the microchiome. We have, instead, subsumed the agency associated with past contributions to the microchiome with the agency of the mother. By extending the maternal logic to past offspring, fetal microchimeric cells in microchiomes of lower genetic diversity than average are selected to remain and assist other microchimeric cells. Fetal cells in microchiomes of higher genetic diversity are selected to self-destroy or destroy cells carrying allelic variants other than theirs.

**Figure 4.**
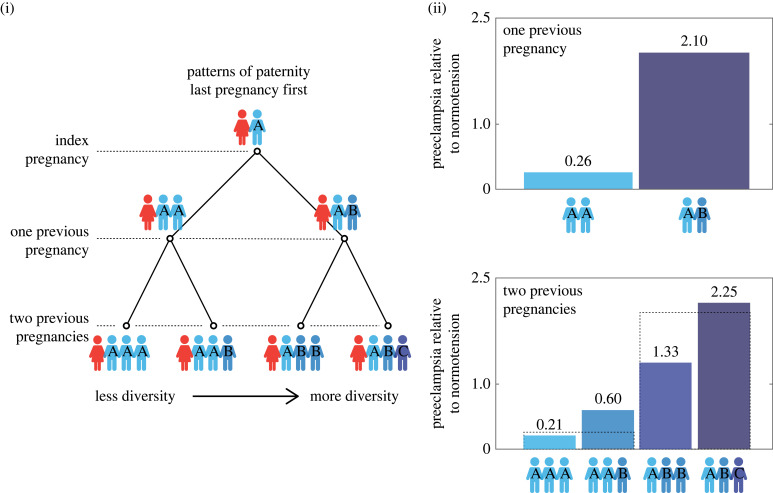
Patterns of paternity and risk of experiencing pre-eclampsia. Panel (i) shows all possible patterns of paternity in three pregnancies. A represents the father of the current pregnancy while B and C are other possible fathers. Thus, pattern AAA corresponds to not changing father in the last two pregnancies while pattern ABC corresponds to changing father at each of the last two pregnancies. Intermediate patterns correspond to having two fathers only, with the same father in current and previous pregnancies in AAB, and the same father in the last two pregnancies in ABB. Panel (ii) shows the risk of pre-eclampsia relative to normal blood pressure corresponding to each paternity pattern. Medical data show a gradual increase in the risk of pre-eclampsia relative to normal blood pressure as the paternity pattern becomes more diverse. Dashed lines correspond to the bars in the 1 pregnancy category to allow comparison. Figure drawn using data from Robillard *et al.* [42].

Our work can explain the observed higher concentration of fetal cells in the bloodstream of mothers with reproductive or autoimmune health problems. Our work predicts that mothers with higher than average diversity of mating partners will have greater numbers of fetal cells and experience greater extraction of resources by the offspring. Greater extraction of resources is expected in conflict scenarios and may result in pre-eclampsia, gestational diabetes, miscarriages and pre-term birth [23,41]. Hence our model predicts greater concentration of fetal cells in the bloodstream of mothers diagnosed with pre-eclapmsia. Medical research does find greater concentration of fetal cells in mothers with pre-eclampsia [14–16]. Furthermore, our model predicts that mothers with higher diversity of mating partners are more likely to experience pre-eclampsia than mothers with lower diversity. Medical research supports our predictions: mothers are ten times more likely to suffer pre-eclampsia in the current pregnancy when changing mating partners relative to when not changing [42–44] (figure 4). Our work also predicts that mothers with higher diversity of mating partners will mount a stronger immune response to the greater presence of fetal cells. Hence our model predicts a greater concentration of fetal cells in the bloodstream of mothers diagnosed with autoimmune diseases, as has been reported by medical researchers, though conflicting results exist [11,13]. Note that we argue that fetal cells should be one of the many contributing factors to these complex disorders.

**Figure 5.**
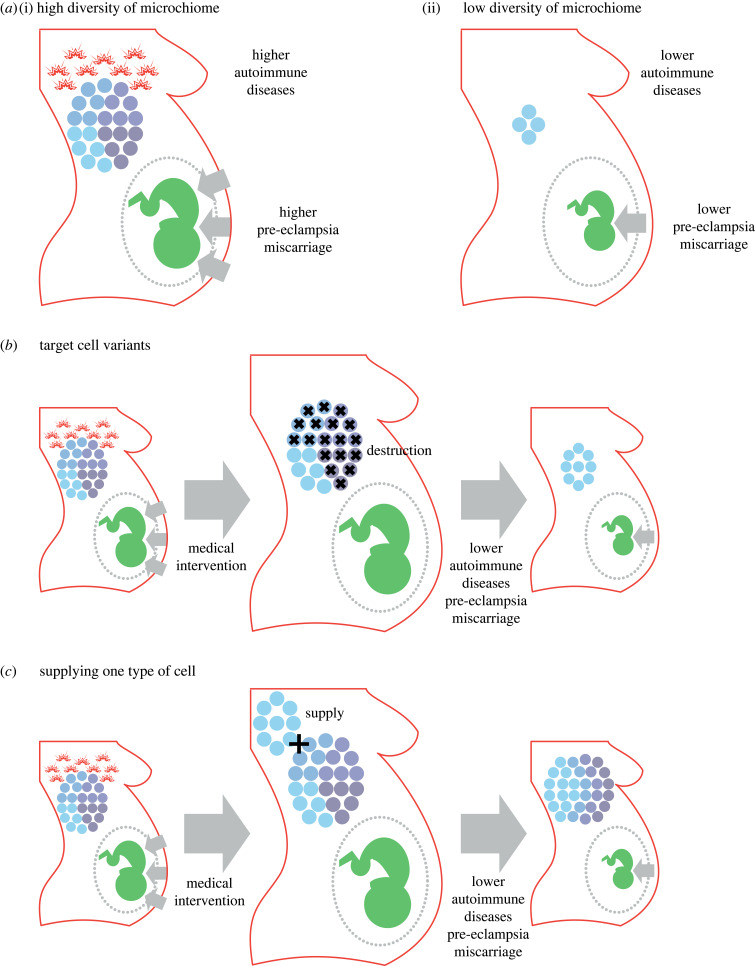
Outline of predicted outcomes of medical interventions. Panel (*a*) summarizes the predicted association between diversity of the microchiome and medical conditions. Panel (*b*) shows the effect of a medical intervention that reduces the genetic diversity of the microchiome by destroying all cell variants except one. Panel (*c*) shows the effect of a medical intervention that reduces the genetic diversity of the microchiome by supplying abundant copies of only one type of cell.

Our work suggests the need to control for the genetic diversity of microchiomes in medical research aiming to link fetal cells and disease. We predict that there should be an association between fetal cells and reproductive and autoimmune diseases only in mothers with fetal and/or grandmaternal microchiomes of greater genetic diversity. The lack of control for different types of mothers should lead to conflicting results as is the case in some clinical tests [11,13]. Should our work be validated by empirical results, it would provide a way of predicting the likelihood of having high-risk pregnancies and/or developing autoimmune diseases. This likelihood could be established by simple analysis of the genetic diversity of fetal cells in women’s bloodstream (figure 5). Furthermore, our work suggests medical interventions to reduce risks of developing health problems; in particular, medical interventions that reduce the diversity of the microchiome by either destroying variants of fetal cells or supplying the same fetal cell (figure 5).

## 4. Methods

### (a) Optimal resource extraction and relatedness

Consider a mother and its offspring. The offspring must extract resources from its mother to survive to maturity. By extracting resources, though, the offspring detracts from the fitness of the future offspring produced by its mother. Let *x* denote the amount of resources the offspring extracts from its mother, let *b*(*x*) denote the fitness benefit the offspring receives by extracting these resources, and let *c*(*x*) denote the total fitness cost paid by all future offspring expected to be produced by the mother.

To model the evolution of resource extraction, we introduce the ‘inclusive-fitness function’ of a focal individual. This function is defined as

4.1 f (x,ρ)=b(x)−ρ c(x),

where *ρ* represents the relatedness between the focal individual and the future offspring of the mother, expressed relative to the relatedness between the focal individual and the present offspring.

The optimum resource-extraction strategy, from the perspective of a focal individual, satisfies *b*′(*x*) − *ρc*′(*x*) = 0. When the focal individual is the mother, we have *ρ* = 1; in the main text, we express *f*(*x*, 1) as *f*_*m*_(*x*). It follows that the optimal strategy from the mother’s perspective, call it $x_{m}^{*}$, satisfies

4.2 $$b^{\prime }(x_{m}^{*})-c^{\prime }(x_{m}^{*})=0\;\text{and}\;b^{\prime \prime }(x_{m}^{*})-c^{\prime \prime }(x_{m}^{*})\leq 0.$$

Henceforth, we assume that the weak inequality (necessary for optimality) in the previous line holds as a strict inequality (sufficient for optimality). When the focal individual is the offspring, *ρ* varies depending on the mother’s diversity of mating partners, and in the main text we write *f*_*o*_(*x*, *ρ*) to emphasize this. In this case, the optimum resource-extraction strategy, call it $x_{o}^{*}$, satisfies

4.3 $$b^{\prime }(x_{o}^{*})-\rho \;c^{\prime }(x_{o}^{*})=0\;\text{and}\;b^{\prime \prime }(x_{o}^{*})-\rho \;c^{\prime \prime }(x_{o}^{*})\leq 0.$$

Again, we assume the weak inequality in the previous line holds strictly.

The equality in (4.3) defines $x_{o}^{*}$ as a function of *ρ*. In recognition of this fact, we write $x_{o}^{*}=x_{o}^{*}(\rho )$. It is important that we understand two features of the geometry of $x_{o}^{*}(\rho )$.

First, implicit differentiation of the equality in (4.3) gives us

4.4 $$x_{o}^{*\prime }(\rho )=\frac{c^{\prime }(x_{o}^{*}(\rho ))}{b^{\prime \prime }(x_{o}^{*}(\rho ))-\rho c^{\prime \prime }(x_{o}^{*}(\rho ))}.$$

The sign of $x_{o}^{*}(\rho )$ is opposite that of $c^{\prime }(x_{o}^{*}(\rho ))$, owing to the fact that the denominator in equation (4.4) is negative. Condition (4.4) makes good biological sense in the context of parent–offspring conflict provided we assume *c*′(*x*) > 0. In that case, as *ρ* grows, the offspring places greater value on the future offspring of its mother, and so $x_{o}^{*}(\rho )$ decreases. What may not be immediately apparent is that $x_{m}^{*}\leq x_{o}^{*}(\rho )$ with equality if and only if *ρ* = 1. In other words, the offspring never wants to extract fewer resources than the mother is willing to give. Henceforth, we assume that the offspring has control of resource extraction and so its optimum is the realized outcome.

Second, if we differentiate (4.4) with respect to *ρ* we arrive at

4.5 $$x_{o}^{*\;\prime \prime }(\rho )=\left(\frac{2\;c^{\prime \prime }(x_{o}^{*}(\rho ))}{c^{\prime }(x_{o}^{*}(\rho ))}-\frac{b^{\prime \prime \prime }(x_{o}^{*}(\rho ))-\rho c^{\prime \prime \prime }(x_{o}^{*}(\rho ))}{b^{\prime \prime }(x_{o}^{*}(\rho ))-\rho c^{\prime \prime }(x_{o}^{*}(\rho ))}\right){\left(\frac{c^{\prime }(x_{o}^{*}(\rho ))}{b^{\prime \prime }(x_{o}^{*}(\rho ))-\rho c^{\prime \prime }(x_{o}^{*}(\rho ))}\right)}^{2}.$$

If the previous expression is positive, then $x_{o}^{*}(\rho )$ is a convex function; if it is negative, then $x_{o}^{*}(\rho )$ is a concave function; if it is zero, then $x_{o}^{*}(\rho )$ is a linear function. Biologically, the convexity/concavity of $x_{o}^{*}(\rho )$ tells us how the level of resource extraction changes with the offspring’s ability to assess its relatedness to future offspring produced by its mother. Following Faria & Gardner’s [33] application of Jensen’s inequality, if $\bar{r}$ is a weighted average of relatedness coefficients—say $\sum_{i}p_{i}\;r_{i}$ for weights *p*_*i*_ and coefficients *r*_*i*_—then we can assert three things:

— if $x_{o}^{*}(\rho )$ is convex, then the resources extracted by an offspring who cannot assess relatedness, $x_{o}(\bar{r})$, are fewer than those extracted by the average offspring who can, $\sum_{i}p_{i}x_{o}(r_{i})$;
— if $x_{o}^{*}(\rho )$ is concave, then the resources extracted by an offspring who cannot assess relatedness, $x_{o}(\bar{r})$, exceed those extracted by the average offspring who can, $\sum_{i}p_{i}x_{o}(r_{i})$;
— if $x_{o}^{*}(\rho )$ is linear, then there is no difference between the resources extracted by an offspring who cannot assess relatedness, $x_{o}(\bar{r})$, and those extracted by the average offspring who can, $\sum_{i}p_{i}x_{o}(r_{i})$.

It is easy to describe a simple scenario in which $x_{o}^{*}(\rho )$ is convex. Consider, for example, *b*(*x*) = 1 − e^−*x*^ and *c*(*x*) = *αx* for 0 < *α* < 1. In this case, $x_{0}^{*}(\rho )=\ln(1/\alpha \rho )$, which is convex. Constructing a simple scenario in which $x_{o}^{*}(\rho )$ is anything but convex is relatively difficult. Intuition says that the optimal level of resource extraction for the offspring ought to be high when relatedness is low. In the biologically artificial (but geometrically relevant) limit as relatedness goes to zero, an optimal offspring would extract infinite resources from its parent, assuming *b*(*x*) is monotonic. It follows that the graph of $x_{o}^{*}(\rho )$ will have a vertical asymptote at *ρ* = 0 in simple cases where *b*(*x*) is monotonic. Of course, the graph of $x_{o}^{*}(\rho )$ should be decreasing toward $x_{m}^{*}$ as *ρ* nears 1. The simplest way to achieve this and include a vertical asymptote at *ρ* = 0 is to posit an example where $x_{o}^{*}(\rho )$ is convex.

The key to constructing a scenario in which $x_{o}^{*}(\rho )$ is not convex, then, is to suppose the benefit of resource extraction declines beyond some point. For example, we might suppose *b*(*x*) = *x*(*α* − *x*) where *α* > 1 now reflects a maximal benefit. If, in addition, *c*(*x*) = *x*, then $x_{o}^{*}(\rho )=(\alpha -\rho )/2$, which is linear. If *b*(*x*) = *x*^2^(*α* − *x*) for $\alpha >\sqrt{3}$ and *c*(*x*) = *x*, we obtain $x_{o}^{*}(\rho )=\left(\alpha +\sqrt{\alpha^{2}-3\rho }\right)/3$, which is concave.

### (b) Offspring’s estimate of relatedness

Now suppose that each mother is committed to a particular diversity of mating partners, *s*, and let *P*(*s*) be the distribution of this diversity strategy in the population (a cumulative distribution function, or cdf). We assume the set of possible strategies, *S*, can be ordered. At one extreme, a mother’s strategy might be ‘always self’ so that *ρ* = 1 from the offspring’s perspective. At the other extreme, a mother’s strategy might be ‘choose a new mate every year’ so that *ρ* = 1/4 from the offspring’s perspective. Between the two extremes, we might find a mother whose strategy is ‘outcross with the same male every year’ so that *ρ* = 1/2 from the offspring’s perspective. To capture the relationship between *s* and *ρ*, we write *ρ* = *r*(*s*) to denote the average relatedness between the offspring and the future offspring produced by its mother given the diversity of her mating partners. For later use, we introduce $\bar{r}=\int_{S}r(s)\;dP(s)$ as the expected relatedness between an offspring and the future offspring produced by its mother.

An offspring can assess its mother’s strategy with varying accuracy. Consider an offspring who assesses with accuracy *a* and whose mother uses mating strategy *s*. Let $P(\hat{s}|s,a)$ be offspring’s estimate of the probability of the event that the mother’s strategy is at least $\hat{s}$ (a conditional cdf). As the offspring’s ability to assess becomes increasingly inaccurate, $P(\hat{s}|s,a)$ tends to $P(\hat{s})$ for all *s*; as the offspring’s ability to assess becomes increasingly accurate, $P(\hat{s}|s,a)$ tends to $H(\hat{s}-s)$, where *H* is the right-continuous Heaviside step function. A simple model for the offspring’s estimate, therefore, is

4.6 $$P(\hat{s}|s,a)=(1-a)P(\hat{s})+aH(\hat{s}-s),$$

where *a* is treated as a real number such that 0 ≤ *a* ≤ 1.

An offspring uses $P(\hat{s}|s,a)$, in turn, to estimate its relatedness to future offspring produced by its mother. Specifically, the estimate it makes is

4.7 $$\hat{r}(s,a)=\int_{S}r(\hat{s})\;dP(\hat{s}|s,a),$$

which, given the simple model in equation (4.6), becomes

4.8 $$\hat{r}(s,a)=(1-a)\int_{S}r(\hat{s})\;dP(\hat{s})+a\int_{S}r(\hat{s})\;dH(\hat{s}-s)=(1-a)\;\bar{r}+a\;r(s).$$

### (c) Inclusive-fitness consequences of improved accuracy

If the offspring uses $\hat{r}(s,a)$ to inform its resource-extraction behaviour, then we predict $x_{o}^{*}(\hat{r}(s,a))=x_{o}^{*}\circ \hat{r}(s,a)$ is the amount of resources it extracts from its mother. In this case,

4.9 $$f\;(x_{o}^{*}\circ \hat{r}(s,a),1)=b(x_{o}^{*}\circ \hat{r}(s,a))-c(x_{o}^{*}\circ \hat{r}(s,a))\;$$

describes the inclusive fitness of the mother, and

4.10 $$f\;(x_{o}^{*}\circ \hat{r}(s,a),r(s))=b(x_{o}^{*}\circ \hat{r}(s,a))-r(s)\;c(x_{o}^{*}\circ \hat{r}(s,a))\;$$

describes the inclusive fitness of the offspring.

All else being equal, selection favours upregulation of any offspring trait that improves the accuracy with which it assesses the mating strategy of its mother. To see why, consider

4.11 $$\begin{array}{rl} \partial_{a}\;f\; & \left(x_{o}^{*}\circ \hat{r}(s,a),r(s)\right)\\ & =(b^{\prime }(x_{o}^{*}\circ \hat{r}(s,a))-r(s)\;c^{\prime }(x_{o}^{*}\circ \hat{r}(s,a)))\;x_{o}^{*\prime }(\hat{r}(s,a))(r(s)-\bar{r})\\ & =(\hat{r}(s,a)-r(s))\;c^{\prime }(x_{o}^{*}\circ \hat{r}(s,a))\;x_{o}^{*\prime }(\hat{r}(s,a))\;(r(s)-\bar{r}), \end{array}$$

where we have used equation (4.3) to simplify the expression. If *r*(*s*) is greater than $\bar{r}$, then equation (4.8) tells us that $\hat{r}(s,a)$ is less than *r*(*s*). On the other hand, if *r*(*s*) is less than $\bar{r}$, then equation (4.8) tells us that $\hat{r}(s,a)$ is greater than *r*(*s*). In both cases, $\left(\hat{r}(s,a)-r(s)\right)$ and $\left(r(s)-\bar{r}\right)$ have opposite signs and so their product is negative. Since *c*′ and $x_{o}^{*\prime }$ also have opposite signs, we can be sure the sign of the derivative in equation (4.11) is positive.

All else being equal, selection favours upregulation of a maternal trait that improves the offspring’s accuracy whenever $r(s)>\bar{r}$ and disfavours upregulation whenever $r(s)<\bar{r}$. To see why consider

4.12 $$\begin{array}{rl} \partial_{a}\;f\; & \left(x_{o}^{*}\circ \hat{r}(s,a),1\right)\\ & =(b^{\prime }(x_{o}^{*}\circ \hat{r}(s,a))-c^{\prime }(x_{o}^{*}\circ \hat{r}(s,a)))\;x_{o}^{*\prime }(\hat{r}(s,a))(r(s)-\bar{r})\\ & =(\hat{r}(s,a)-1)\;c^{\prime }(x_{o}^{*}\circ \hat{r}(s,a))\;x_{o}^{*\prime }(\hat{r}(s,a))\;(r(s)-\bar{r}), \end{array}$$

where we have, again, used equation (4.3) to simplify. The sign of $\left(\hat{r}(s,a)-1\right)$ is negative; the sign of *c*′ is positive; the sign of $x_{o}^{*\prime }(\hat{r}(s,a))$ is negative. Therefore, the sign of the derivative in equation (4.12) is determined solely by the sign of $r(s)-\bar{r}$. When $r(s)-\bar{r}$ is positive the derivative is also positive, and when $r(s)-\bar{r}$ is negative the derivative is also negative.

### (d) Microchimeric cells as a source of information

Now suppose accuracy *a* is determined by microchimeric cells, with increased numbers of cells improving *a* and decreased numbers reducing *a*. Let *Y* be a trait expressed by the offspring that increases the number of microchimeric cells when upregulated. Let *Z* be a trait expressed by the mother that decreases the number of microchimeric cells when upregulated. Finally, let *χ*(*y*, *z*) = *a* be the accuracy of the assessment made by an offspring who expresses *Y* at level *y* when its mother expresses *Z* at level *z*.

We now introduce $w_{m}(y,z)=f\;(x_{o}^{*}\circ \hat{r}(s,\chi (y,z)),1)$ to represent the inclusive-fitness of the mother and $w_{o}(y,z)=$ $f\;(x_{o}^{*}\circ \hat{r}(s,\chi (y,z)),r(s))$ to represent the inclusive-fitness of the offspring. With this new notation, we describe the selection gradient acting on *y* as ∂*w*_*o*_/∂*y* and that acting on *z* as ∂*w*_*m*_/∂*z*. From equations (4.11) and (4.12), we know that

4.13 $$r(s)>\bar{r}\;\text{implies}\;\frac{\partial w_{o}}{\partial y}>0\;\text{and}\;\frac{\partial w_{m}}{\partial z}<0,$$

and so selection increases *χ*, i.e. selection improves offspring accuracy. From the same equations, we also know that

4.14 $$r(s)<\bar{r}\;\text{implies}\;\frac{\partial w_{o}}{\partial y}>0\;\text{and}\;\frac{\partial w_{m}}{\partial z}>0,$$

and so mother and offspring are in conflict over *χ*, i.e. mother strives for fewer cells and, therefore, lower accuracy, while the offspring strives for more cells and, therefore, greater accuracy. Of course, if $r(s)=\bar{r}$ then equations (4.11) and (4.12) tell us that both mother and offspring are indifferent to cell numbers.

If a mother cannot express *z* conditional upon *r*(*s*), then whether she strives for more or fewer cells depends on the expected value of equation (4.12). Specifically, it depends on the expected value of

4.15 $$\begin{array}{rl} (\hat{r}(s,\chi (y,z))-1)\; & c^{\prime }(x_{o}^{*}\circ \hat{r}(s,\chi (y,z)))\\ & x_{o}^{*\prime }(\hat{r}(s,\chi (y,z)))\;(r(s)-\bar{r})\cdot \partial_{z}\;\chi (y,z), \end{array}$$

where the expectation is taken with respect to the distribution of reproductive value over *s* values. If the expectation is positive, then the mother who cannot condition on *s* will strive to eliminate cells. If the expectation is negative, then the mother who cannot condition on *s* will encourage cells. If the expectation is zero, then the mother who cannot condition on *s* will be indifferent to the presence of cells.

Using equation (4.8) and *a* = *χ*(*y*, *z*), we rewrite (4.15) as

4.16 $$\begin{array}{rl} (a\;(r-\bar{r})-(1-\bar{r}))\; & c^{\prime }(x_{o}^{*}((1-a)\;\bar{r}+a\;r))\\ & x_{o}^{*\prime }((1-a)\;\bar{r}+a\;r)\;(r-\bar{r})\cdot \partial_{z}\;\chi (y,z). \end{array}$$

We have written *r* = *r*(*s*) because we can change variable and take the expectation with respect to the distribution of *r* values. If $r=\bar{r}+\varepsilon$, where ε has mean zero and variance $\sigma_{r}^{2}$, then we can find the change in maternal inclusive fitness due to increased upregulation of *z* to be

4.17 $$\begin{array}{rl} (a(x_{o}^{*\prime }(\bar{r})-(1-\bar{r})x_{o}^{*\prime \prime }(\bar{r}))c^{\prime }(x_{o}^{*}(\bar{r}))\;-(1-\bar{r})\;{(x_{o}^{*\prime }(\bar{r}))}^{2}\;c^{\prime \prime } & (x_{o}^{*}(\bar{r})))\\ & \sigma_{r}^{2}\;\partial_{z}\;\chi (y,z). \end{array}$$

If *c*(*x*) is linear and increasing, and *b*(*x*) is monotonic and constructed so that x*(*ρ*) is convex (e.g. the example in §4a), then (4.17) shows that the expected change in maternal inclusive fitness is positive. As mentioned, this implies the mother eliminates cells. Biologically, this makes sense: the convex geometry of $x_{o}^{*}(\rho )$ means that by keeping relatedness information from offspring a mother is able to encourage offspring to extract fewer resources than they would have otherwise. If *c*(*x*) is linear and increasing, and *b*(*x*) is non-monotonic and constructed so that $x_{o}^{*}(\rho )$ is either linear or concave (e.g. the examples in §4a), then (4.17) can evaluate to a positive quantity. When x*_o_(*ρ*) is concave, a mother strives to provide information to offspring because it encourages them to extract fewer resources. Offspring resource extraction does not change with or without information when $x_{o}^{*}(\rho )$ is linear. Information in this case might be viewed as bet-hedging.

## Data Availability

This article has no additional data.

## References

[1] Dawe GS, Tan XW, Xiao Z-C. 2007 Cell migration from baby to mother. Cell Adhes. Migr. **1**, 19-27. (10.4161/cam.4082) PMC2633676 19262088

[2] Boddy AM, Fortunato A, Sayres MW, Aktipis A. 2015 Fetal microchimerism and maternal health: a review and evolutionary analysis of cooperation and conflict beyond the womb. Bioessays **37**, 1106-1118. (10.1002/bies.201500059) 26316378 PMC4712643

[3] Gammill HS, Harrington E. 2017 Microchimerism: defining and redefining the prepregnancy context: a review. Placenta **60**, 130-133. (10.1016/j.placenta.2017.08.071) 28911790 PMC5718967

[4] Lo Y, Lau T, Chan L, Leung T, Chang A. 2000 Quantitative analysis of the bidirectional fetomaternal transfer of nucleated cells and plasma DNA. Clin. Chem. **46**, 1301-1309. (10.1093/clinchem/46.9.1301) 10973858

[5] Kinder JM, Stelzer IA, Arck PC, Way S. 2017 Immunological implications of pregnancy-induced microchimerism. Nat. Rev. Immunol. **17**, 483-494. (10.1038/nri.2017.38) 28480895 PMC5532073

[6] Bianchi D, Zickwolf G, Weil G, Sylvester S, DeMaria M. 1996 Male fetal progenitor cells persist in maternal blood for as long as 27 years postpartum. Proc. Natl Acad. Sci. USA **93**, 705-708. (10.1073/pnas.93.2.705) 8570620 PMC40117

[7] Gammill HS, Adams Waldorf KM, Aydelotte TM, Lucas J, Leisenring WM, Lambert NC, Nelson JL. 2011 Pregnancy, microchimerism, and the maternal grandmother. PLoS ONE **6**, e24101. (10.1371/journal.pone.0024101) 21912617 PMC3166068

[8] Karlmark KR et al. 2021 Grandmaternal cells in cord blood. eBiomedicine **74**, 103721. (10.1016/j.ebiom.2021.103721) 34844192 PMC8720789

[9] Kolialexi A, Tsangaris G, Antsaklis A, Mavrou A. 2004 Rapid clearance of fetal cells from maternal circulation after delivery. Ann. N. Y. Acad. Sci. **1022**, 113-118. (10.1196/annals.1318.018) 15251948

[10] Fujiki Y, Johnson KL, Tighiouart H, Peter I, Bianchi DW. 2008 Fetomaternal trafficking in the mouse increases as delivery approaches and is highest in the maternal lung. Biol. Reprod. **79**, 841-848. (10.1095/biolreprod.108.068973) 18633138 PMC2714997

[11] Nelson JL. 2012 The otherness of self: microchimerism in health and disease. Trends Immunol. **33**, 421-427. (10.1016/j.it.2012.03.002) 22609148 PMC3516290

[12] Forsberg LA, Gisselsson D, Dumanski JP. 2017 Mosaicism in health and disease: clones picking up speed. Nat. Rev. Genet. **18**, 128-142. (10.1038/nrg.2016.145) 27941868

[13] Fjeldstad HES, Johnsen GM, Staff AC. 2020 Fetal microchimerism and implications for maternal health. Obstet. Med. **13**, 112-119. (10.1177/1753495X19884484) 33093862 PMC7543167

[14] Holzgreve W, Ghezzi F, Edoardo Di Naro MD, Maymon E. 1998 Disturbed feto-maternal cell traffic in preeclampsia. Obstet. Gynecol. **91**, 669-672. (10.1016/S0029-7844(98)00068-4) 9572208

[15] Gammill HS, Aydelotte TM, Guthrie KA, Nkwopara EC, Nelson JL. 2013 Cellular fetal microchimerism in preeclampsia. Hypertension **62**, 1062-1067. (10.1161/HYPERTENSIONAHA.113.01486) 24101661 PMC4395136

[16] McCartney SA, Kolarova T, Kanaan SB, Chae A, Laughney CI, Nelson JL, Gammill HS, Shree R. 2023 Increased fetal microchimerism in immune and stem cell subsets in preeclampsia. Am. J. Reprod. Immunol. **89**, e13666. (10.1111/aji.13666) 36482289 PMC10413445

[17] Leung T, Zhang J, Lau T, Hjelm N, Lo Y. 1998 Maternal plasma fetal DNA as a marker for preterm labour. Lancet **352**, 1904-1905. (10.1016/S0140-6736(05)60395-9) 9863792

[18] Peterson S, Nelson J, Gadi V, Gammill H. 2013 Fetal cellular microchimerism in miscarriage and pregnancy termination. Chimerism **4**, 136-138. (10.4161/chim.24915) 23723084 PMC3921195

[19] Gammill HS, Stephenson MD, Aydelotte TM, Nelson JL. 2014 Microchimerism in recurrent miscarriage. Cell. Mol. Immunol. **11**, 589-594. (10.1038/cmi.2014.82) 25242272 PMC4220842

[20] Haig D. 2014 Interbirth intervals, intrafamilial, intragenomic and intrasomatic conflict. Evol. Med. Public Health **2014**, 12-17. (10.1093/emph/eou002) 24480612 PMC3917425

[21] Haig D. 2014 Does microchimerism mediate kin conflicts? Chimerism **5**, 53-55. (10.4161/chim.29122) 24810968 PMC4199807

[22] Trivers RL. 1974 Parent-offspring conflict. Am. Zool. **14**, 249-264. (10.1093/icb/14.1.249)

[23] Haig D. 1993 Genetic conflicts in human pregnancy. Q. Rev. Biol. **68**, 495-532. (10.1086/418300) 8115596

[24] Patten MM. 2021 On being a monkey’s uncle: germline chimerism in the Callitrichinae and the evolution of sibling rivalry. Am. Nat. **197**, 502-508. (10.1086/713110) 33755537

[25] Hamilton WD. 1964 The genetical evolution of social behaviour. I and II. J. Theor. Biol. **7**, 1-52. (10.1016/0022-5193(64)90038-4) 5875341

[26] Alberts B, Johnson A, Lewis J, Raff M, Roberts K, Walter P. 2002 Molecular biology of the cell. New York, NY: Garland Science.

[27] Grafen A. 2007 Detecting kin selection at work using inclusive fitness. Proc. R. Soc. B **274**, 713-719. (10.1098/rspb.2006.0140) PMC2197210 17254996

[28] Grafen A. 2009 Formalizing Darwinism and inclusive fitness theory. Phil. Trans. R. Soc. B **364**, 3135-3141. (10.1098/rstb.2009.0056) 19805422 PMC2781868

[29] West SA, Gardner A. 2013 Adaptation and inclusive fitness. Curr. Biol. **23**, R577-R584. (10.1016/j.cub.2013.05.031) 23845249

[30] Gardner A, Úbeda F. 2017 The meaning of intragenomic conflict. Nat. Ecol. Evol. **1**, 1807-1815. (10.1038/s41559-017-0354-9) 29109471

[31] Haig D. 1996 Placental hormones, genomic imprinting, and maternal-fetal communication. J. Evol. Biol. **9**, 357-380. (10.1046/j.1420-9101.1996.9030357.x)

[32] Wilkins J, Haig D. 2001 Genomic imprinting of two antagonistic loci. Proc. R. Soc. Lond. B **268**, 1861-1867. (10.1098/rspb.2001.1651) PMC1088820 11564340

[33] Faria G, Gardner A. 2020 Does kin discrimination promote cooperation? Biol. Lett. **16**, 20190742. (10.1098/rsbl.2019.0742) 32183635 PMC7115181

[34] Godfray H. 1995 Evolutionary-theory of parent-offspring conflict. Nature **376**, 133-138. (10.1038/376133a0) 7603563

[35] Antonioli L, Blandizzi C, Pacher P, Guilliams M, Hasko G. 2019 Rethinking communication in the immune system: the quorum sensing concept. Trends Immunol. **40**, 88-97. (10.1016/j.it.2018.12.002) 30611647 PMC6619492

[36] Cherkas L, Oelsner E, Mak Y, Valdes A, Spector T. 2004 Genetic influences on female infidelity and number of sexual partners in humans: a linkage and association study of the role of the vasopressin receptor gene (AVPR1A). Twin Res. **7**, 649-658. (10.1375/twin.7.6.649) 15607016

[37] Forstmeier W, Martin K, Bolund E, Schielzeth H, Kempenaers B. 2011 Female extrapair mating behavior can evolve via indirect selection on males. Proc. Natl Acad. Sci. USA **108**, 10 608-10 613. (10.1073/pnas.1103195108) PMC3127899 21670288

[38] Zietsch BP, Westberg L, Santtila P, Jern P. 2015 Genetic analysis of human extrapair mating: heritability, between-sex correlation, and receptor genes for vasopressin and oxytocin. Evol. Hum. Behav. **36**, 130-136. (10.1016/j.evolhumbehav.2014.10.001)

[39] Murray DR, Gildersleeve KA, Fales MR, Haselton MG. 2017 MHC homozygosity is associated with fast sexual strategies in women. Adapt. Hum. Behav. Physiol. **3**, 101-117. (10.1007/s40750-016-0057-5)

[40] Dush CMK, Arocho R, Mernitz S, Bartholomew K. 2018 The intergenerational transmission of partnering. PLoS ONE **13**, e0205732. (10.1371/journal.pone.0205732) 30422991 PMC6233917

[41] Wilkins JF. 2011 Genomic imprinting and conflict-induced decanalization. Evolution **65**, 537-553. (10.1111/j.1558-5646.2010.01147.x) 21029079

[42] Robillard PY, Hulsey TC, Alexander GR, Keenan A, de Caunes F, Papiernik E. 1993 Paternity patterns and risk of preeclampsia in the last pregnancy in multiparae. J. Reprod. Immunol. **24**, 1-12. (10.1016/0165-0378(93)90032-D) 8350302

[43] Robillard PY, Périanin J, Janky E, Miri EH, Hulsey TC, Papiernik E. 1994 Association of pregnancy-induced hypertension with duration of sexual cohabitation before conception. Lancet **344**, 973-975. (10.1016/S0140-6736(94)91638-1) 7934427

[44] Hercus A, Dekker G, Leemaqz S. 2020 Primipaternity and birth interval; independent risk factors for preeclampsia. J. Matern.-Fetal Neonatal Med. **33**, 303-306. (10.1080/14767058.2018.1489794) 29914280

